# Prevalence of *Prototheca* spp. on dairy farms in Poland – a cross‐country study

**DOI:** 10.1111/1751-7915.13394

**Published:** 2019-03-19

**Authors:** Tomasz Jagielski, Henryk Krukowski, Mariola Bochniarz, Tomasz Piech, Katarzyna Roeske, Zofia Bakuła, Łukasz Wlazło, Piotr Woch

**Affiliations:** ^1^ Department of Applied Microbiology Institute of Microbiology Faculty of Biology University of Warsaw I. Miecznikowa 1 02‐096 Warsaw Poland; ^2^ Department of Microbiology and Reproductive Biology University of Life Sciences Akademicka 13 20‐950 Lublin Poland; ^3^ Department and Clinic of Animal Reproduction Faculty of Veterinary Medicine University of Life Sciences Głęboka 30 20‐612 Lublin Poland; ^4^ Department of Animal Hygiene and Environment University of Life Sciences Akademicka 13 20‐950 Lublin Poland; ^5^ Regional Agrochemical Station in Lublin Sławinkowska 5 20‐810 Lublin Poland

## Abstract

The *Prototheca* algae have recently emerged as an important cause of bovine mastitis globally. Here, we present results of a first large‐scale, cross‐country survey on the prevalence of *Prototheca* spp. in dairy cows, and their environment in Poland. A total of 1211 samples were collected and microbiologically analysed. Included within this number were milk (*n *=* *638), body swabs (*n *=* *374) and environmental samples (*n *=* *199), originating from 400 dairy cows and their surroundings, on 16 dairy farms, based in all major provinces of the country. *Prototheca* spp. were the third, after *Streptococcus* and *Staphylococcus* spp., most common mastitis pathogens. The overall prevalence of protothecal mastitis was 8.3% (33/400), with the majority (75.8%) of cases having a subclinical course, and all but one attributable to *P. zopfii* genotype 2. *Prototheca* spp. were cultured from body swabs of both healthy and mastitic cows, yet the isolation rate among the latter was conspicuously lower (12.3% vs. 17.8%). Forty‐two (21.2%) environmental samples yielded growth of *Prototheca* spp. However, no clear association between *Prototheca* mastitis in dairy cows and the algal isolation from the herd environment was found. Nor was there any association between the environmental recovery of the algae and farm management practices.

## Introduction

Mastitis is the single most common and costly disease of dairy cattle worldwide. The economic impact of mastitis is due to a decreased milk production, elevated costs of veterinary services and treatment and premature culling of infected animals (Seegers *et al*., 2003). The annual financial losses from clinical mastitis (CM) have been calculated at 1.5 billion Euro in the European Union (EU; Food Safety Authority) or up to 182 Euro per cow (Huijps *et al*., 2008). However, the overall economic burden of mastitis is much greater, since CM is only a small fraction of the problem, with subclinical mastitis (SCM), an asymptomatic form of the disease, being up to 40 times more common.

Bovine mastitis is a complex disorder, with a number of factors influencing the epidemiological dynamics and transmission of the disease, its clinical symptomatology and treatment response. All these factors, either of pathogen, host or environmental origin interplay differently in this triad relationship to produce disease. The complexity of mastitis is mirrored by a wide spectrum of causative agents, epidemiologically categorized as contagious or environmental, with their primary reservoir being an infected udder and cow's surroundings respectively (Contreras and Rodriguez, 2011). The implementation of long‐standing control programs for contagious mastitis led to a shift in the prevalence of mastitis pathogens towards environmental bacteria, including the coliforms (*Escherichia coli*,* Klebsiella* spp., *Citrobacter* spp., *Enterobacter* spp.), coagulase‐negative staphylococci (CNS), streptococci (*Streptococcus uberis*,* Streptococcus dysgalactiae*) and enterococci (*Enterococcus faecalis*,* Enterococcus faecium*), as well as non‐bacterial organisms, such as fungi, mostly yeasts and achlorophyllous, yeast‐like algae of the *Prototheca* genus (Zadoks and Fitzpatrick, 2009; Contreras and Rodriguez, 2011). The *Prototheca* algae, whose aetiological relation to the mastitis was first contested and then heavily underestimated, either due to misidentification as yeasts or dismissal as contaminating, saprophytic microflora, have emerged as a significant cause of bovine mastitis globally, with the prevalence steadily increasing over the last two decades. Since 1952, when the first case of *Prototheca* mastitis was described by Lerche (1952), the disease has been reported in most of the countries with commercial dairy farming and highly developed milk industries, including the United States, Canada, Belgium, France, Denmark, Germany, Italy, Brazil, China, Japan and New Zealand (Hodges *et al*., 1985; Anderson and Walker, 1988; Lagneau, 1996; Aalbaek *et al*., 1998; Buzzini *et al*., 2004; Bueno *et al*., 2006; Moller *et al*., 2007; Aouay *et al*., 2008; Osumi *et al*., 2008; Ricchi *et al*., 2010; Gao *et al*., 2012; Pieper *et al*., 2012; Sobukawa *et al*., 2012; Bozzo *et al*., 2014; Shahid *et al*., 2016).

Until now, two of the eight known *Prototheca* species have been implicated in bovine mastitis, namely *P. zopfii* and *P. blaschkeae*, with the former being responsible for the bulk of the cases. Noteworthy, of the two genotypes, into which *P. zopfii* is divided (Roesler *et al*., 2006), only genotype 2 has been isolated from the affected animals (Moller *et al*., 2007; Aouay *et al*., 2008; Marques *et al*., 2008; Osumi *et al*., 2008; Ricchi *et al*., 2010, 2013; Jagielski *et al*., 2011; Gao *et al*., 2012; Sobukawa *et al*., 2012; Bozzo *et al*., 2014; Shahid *et al*., 2016). *Prototheca zopfii* genotype 1 has produced subclinical mastitis in a cow, only following an experimental challenge (Ito *et al*., 2011).

*Prototheca* mastitis most often presents as a chronic process characterized by a permanent increase in somatic cell count and dramatic decline in milk yield (Janosi *et al*., 2001a). The inflammatory response is usually mild with a progressive damage to mammary parenchyma and concurrent alveolar atrophy (Janosi *et al*., 2001a). A hallmark of *Prototheca* infections is their refractoriness to most of the therapeutic regimens currently available in veterinary medicine. Resistance to conventional therapy (Buzzini *et al*., 2008; Jagielski *et al*., 2012) together with extremely low self‐recovery rates, advocates culling as the preferred option to contain and prevent the infection from spreading within the herds (Janosi *et al*., 2001a). Furthermore, the ability of *Prototheca* spp. to survive standard chlorination or a wide range of temperatures, including pasteurizing temperatures (Marques *et al*., 2010; Lassa *et al*., 2011), contributes to the environmental persistence of the algae, which, in turn, may increase the risk of their transmission to animals.

Despite the rising awareness of *Prototheca* mastitis and the risks it poses to the dairy sector, systematic and comprehensive studies on the epidemiology of algal infections in dairy populations are fragmentary and based primarily on regional experiences. The purpose of this work was to investigate the occurrence of *Prototheca* algae on 16 dairy farms, representing all major provinces of Poland. The present study is thus the first nationwide survey for the bovine mammary protothecosis in Poland.

## Results

### Management and sanitary conditions on the farms

The location of 16 dairy farms investigated in this study was shown in Fig. S1. These farms housed a total of 2829 lactating cows, mostly of the Holstein‐Friesian breed, but also of the Simmental (VI), Polish Red (IX) or Montbéliarde (XV) breed. The mean number of cows in a herd was 176.8 ± 152.1 (range, 19–584). The average 305‐day milk production per cow was 8138.3 l ± 1926.9 (range, 4500–11 986.3 l), whereas the average annual milk production per herd was 16 600 hl ± 12 299.5 (range, 860–70 000). The mean slaughter rate was 27% ± 5.3 (range, 15–36%), the majority due to mastitis and fertility disorders (Table S1).

Data on the management and sanitary practices on the dairy farms under the study were summarized in Table S1. Most of the farms had a closed herd turnover (75%), free‐stall barns (56.2%) with straw bedding (93.8%), total mixed ration (TMR) feeding (68.8%), and drinking water supplied by municipal water distribution network (100%). The cows had a temporary access to pasture and grazing areas (87.5%) and were all under regular veterinary control (100%). Milking at all farms was done mechanically, in a double‐rowed milking parlour (62.5%) or with a milking pipeline (37.5%), twice daily, by experienced dairy farm milkers. On all farms, aprons and gloves were used regularly by all milking crew members.

Premilking udder preparation was routinely performed on 13 (81.2%) dairy farms and consisted of water hose wash, paper towel manual drying and disinfection prior to machine attachment. All but three farms applied a standard postmilking hygiene practice, which was teat dipping with various iodine‐containing, commercially available disinfectants.

### Mastitis detection

A total of 400 lactating cows and 1550 quarters were examined clinically and by California Mastitis Test (CMT). The test was positive for 243 (60.8%) cows and the proportion of CMT‐positive animals per herd, ranged from 33.3% (dairy farm XI) to 92.9% (II), with a mean of 62.4% ± 17.8 (Table 1).

**Table 1:** Distribution of mastitis pathogens in milk samples and *Prototheca* spp. in body swabs and environmental samples

Sample information	Herd																Total, *n*
	I	II	III	IV	V	VI	VII	VIII	IX	X	XI	XII	XIII	XIV	XV	XVI	
Total animals, *n*	30	14	18	21	30	30	30	26	17	27	30	25	30	27	25	20	400
CMT+ animals, *n* (% total animals)	23 (76.7)	13 (92.9)	9 (50)	18 (85.7)	24 (80)	17 (56.7)	17 (56.7)	20 (76.9)	14 (82.3)	12 (44.4)	10 (33.3)	12 (48)	14 (46.7)	12 (44.4)	16 (64)	12 (60)	243 (60.8)
Total samples (QMS, BS, ES), *n*	101	120	70	79	75	75	103	78	61	62	68	61	54	56	82	66	1211
QMS, *n*	36	48	40	50	47	53	38	54	37	36	32	32	30	29	44	32	638
QMS from CMT‐negative cows, *n*	5	8	12	8	12	12	16	12	12	12	20	12	12	12	12	20	197 (30.9)
QMS from CMT‐positive cows, *n*	31	40	28	42	35	41	22	42	25	24	12	20	18	17	32	12	441 (69.1)
Growth‐positive QMS, *n* (% QMS)	20 (55.6)	35 (72.9)	20 (50)	19 (38)	17 (36.2)	36 (67.9)	17 (44.7)	18 (33.3)	24 (64.9)	19 (52.8)	9 (28.1)	12 (37.5)	2 (6.7)	13 (44.8)	22 (50)	16 (50)	299 (46.9)
*Staphylococcus* sp.	4	0	12	1	5	1	2	1	0	2	3	6	1	4	13	1	56 (8.8)
*Streptococcus* sp.	4	0	5	17	10	34	2	13	24	5	3	2	1	8	1	9	138 (21.6)
*Enterobacteriaceae*	1	0	0	0	0	0	0	0	0	4	0	1	0	0	3	0	9 (1.4)
*Enterococcus* sp.	0	0	0	0	0	0	0	0	0	7	2	3	0	1	0	0	13 (2)
*Corynebacterium* sp.	1	0	0	0	0	1	0	0	0	0	1	0	0	0	1	0	4 (0.6)
Yeasts	0	0	2	0	1	0	1	4	0	1	0	0	0	0	0	5	14 (2.2)
*Prototheca* sp.	10 [6]	35 [12]	1 [1]	1 [1]	1 [1]	0 [0]	12 [7]	0 [0]	0 [0]	0 [0]	0 [0]	0 [0]	0 [0]	0 [0]	4 [4]	1 [1]	65 (10.2)
BS, *n*	48	64	20	20	20	15	45	15	15	12	20	12	12	12	28	16	374
Proto+, *n* (% BS)	17 (35.4)	0	3 (15)	7 (35)	4 (20)	0	14 (31.1)	2 (13.3)	0	2 (16.7)	0	7 (58.3)	0	0	3 (10.7)	0	59 (15.8)
Mouth, *n*	NC	0	1^a^	1	0	0	2^a^	1	0	0	0	3	0	0	0	0	8 (2.1)
Nose, *n*	3	0	0	0	0	0	1	0	0	0	0	3	0	0	0	0	7 (1.9)
Vagina, *n*	1	0	0	1	0	0	4[Link]	0	0	1	0	1	0	0	1	0	9 (2.4)
Rectum, *n*	6^c^	NC	0	2	0	0	6^c^	1	0	1	0	0	0	0	2[Link]	NC	18 (4.8)
Faeces, *n*	7^b^	0	2^a^	3^a^	4^a^	0	1	0	0	NC	NC	NC	NC	NC	NC	0	17 (4.5)
ES, *n*	17	8	10	9	8	7	20	9	9	14	16	17	12	15	10	18	199
Proto+, *n* (% ES)	8 (47.1)	0 (0)	4 (40)	4 (44.4)	0 (0)	0 (0)	13 (65)	1 (11.1)	0 (0)	1 (7.1)	2 (12.5)	5 (29.4)	2 (16.7)	0 (0)	0 (0)	2 (11.1)	42 (21.1)
Building and equipment surfaces, *n*	NC	NC	1 (10)	NC	NC	NC	3 (15)	NC	NC	0	1 (6.3)	0	0	0	0	1 (5.6)	6 (3)
Water, *n*	6 (35.3)	0	1 (10)	4 (44.4)	0	0	1 (5)	1 (11.1)	0	0	0	0	0	0	0	0	13 (6.5)
Bedding, *n*	NC	0	0	NC	NC	NC	3 (15)	NC	NC	0	0	1 (5.9)	2 (16.7)	0	0	NC	6 (3)
Feed, *n*	1 (5.9)	0	1 (10)	0	0	0	0	0	0	0	1 (6.3)	2 (11.8)	0	0	0	1 (5.6)	6 (3)
Mud/soil, *n*	0	0	1 (10)	0	0	0	6 (30)	0	0	0	0	1 (5.9)	0	0	0	0	8 (4)
Manure, *n*	1 (5.9)	0	0	0	0	0	0	0	0	1 (7.1)	0	1 (5.9)	0	0	0	0	3 (1.5)
Total Proto+ samples	35	35	8	12	5	0	39	3	0	3	2	12	2	0	7	3	166 (15.7)

BS, body swabs; CMT+, positive result of California Mastitis Test; ES, environmental samples; NC, not collected; Proto+, *Prototheca* spp. containing sample; QMS, quarter milk samples.

**a.** Including one isolate from a CM/SCM cow.

**b.** Including two isolates from CM/SCM cows.

**c.** Three isolates from CM/SCM cows.

Percentage of samples (%) were given in brackets.

The number of *Prototheca‐*affected animals were given in square brackets.

Overall, 441 (28.5%) quarter milk samples (QMS) from CMT‐positive cows were cultured. Of these samples, 299 (67.8%) from 185 (76.1%) cows were positive for microbial growth (Fig. S2). The proportion of culture positivity differed between the farms, ranging from 28.1% (XI) to 72.9% (II), with a mean of 45.8% ± 16.3.

**Figure 1:**
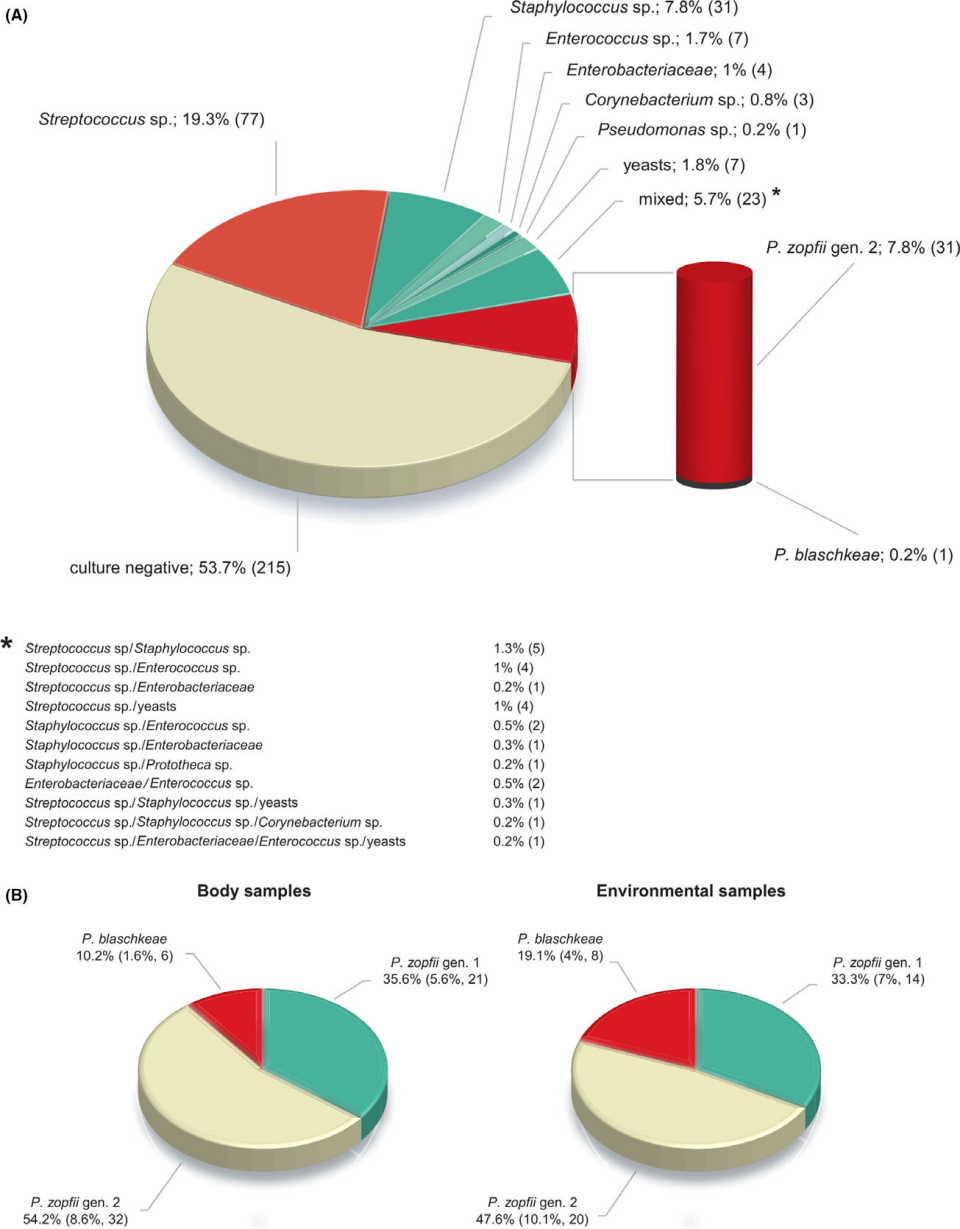
(A) Mastitis aetiology in 185 cows, out of 400 cows tested, based on their milk culture results. (B) Species profiling of *Prototheca* cultures obtained from animal body and environmental samples. Percentages were calculated with reference to either *Prototheca*‐positive samples or all samples collected (in brackets).

The results of culturing for bacterial and yeast mastitis pathogens were described in Supporting Information and Fig. 1A.

*Prototheca* spp. were isolated from 65 (14.7%) QMS collected from 33 (13.6%) CMT‐positive cows at eight dairy farms (I‐V, VII and XV, XVI). Seventeen (51.5%) had single‐quarter infections, while the remaining 16 (48.5%) had multiple‐quarter infections (mean, 2 ± 1.2; mean for CM, 2.5 ± 1.4; mean for SCM, 1.8 ± 1.1). Eight (24.2%) cows suffered from moderate CM (changes in milk and visible signs of inflammation of the udder), while the remaining 25 (75.8%) cows presented with SCM. In all but one cow, the *Prototheca* algae were the sole pathogens cultured from milk samples. Only milk from a single cow yielded growth, albeit from separate QMS, of *Prototheca* spp. and *Staphylococcus aureus*.

The overall cow‐level prevalence of mastitis due to *Prototheca* spp. was 8.3% (33/400), with the within‐herd prevalences ranging from 0% (farms VI, and VIII‐XIV) to 85.7% (II; mean, 10.2% ± 21.5). It is of note, however, that except five farms (III, IV, VI, IX and XI), the herd‐level prevalences might be biased by insufficient sample size, according to statistical simulations (cf. Study design and sample size).

The prevalences of *Prototheca* mastitis among cows with CM and SCM were of 38.1% (8/21) and 11.4% (25/220) respectively.

Cases of CM were more common with an open herd turnover, while cases of SCM occurred more frequently on closed turnover farms (*P *=* *0.035; Cramer's V > 0.4). Farms which furnished quarantine units produced more CM cases, yet less SCM cases, than those without such facilities (*P *=* *0.035; Cramer's *V* > 0.4).

Cows that were culture‐positive for *Prototheca* spp. had their average SCC significantly higher when compared with the control animals (6.2 × 10^6^ vs. 0.2 × 10^6^ cells ml^−1^; *P *<* *0.0001). The average SCC for cows with clinical *Prototheca* mastitis was almost twice that for cows with SCM (9.1 × 10^6^ vs. 4.9 × 10^6^ cells ml^−1^; *P *<* *0.0001). No significant differences between blood cells counts of mastitis and control cows were observed (Table S2).

### *Prototheca* spp. in other than milk samples

Apart from QMS, 374 body samples (incl. stool) were collected from 87 cows, including 33 cows with *Prototheca* mastitis and 54 control animals. A total of 59 *Prototheca* spp. cultures were obtained, most of which (42 or 71.2%) originating from the controls (22 cows). *Prototheca* cultures were from both CM and SCM cows, yet with a heavy skew towards the latter (14 cultures from 11 SCM cows vs. 3 cultures from 3 CM cows).

*Prototheca* spp. in mastitic cows occurred mostly in rectum and faeces (13 isolates from 2 CM and 10 SCM cows). Four isolates were cultured from oral (two isolates from CM and SCM cows) and vaginal (two isolates from SCM cows) swabs.

The frequencies of *Prototheca* spp. isolation among control animals were as follows: 35.3% (12/34) for faeces, 21.7% (10/46) for rectal swabs, 13% (7/54) equally for nasal and vaginal swabs and 12.5% (6/48) for oral swabs (Table S3).

Forty‐two (21.1%) out of 199 environmental samples were positive for *Prototheca* spp. (Table S3). These samples originated from ten dairy farms (I, III, IV, VII, VIII, X‐XIII and XVI), including five (VIII, and X‐XIII) where no *Prototheca* spp. were isolated from milk samples. In two of such farms (XI and XIII) *Prototheca* spp. were also absent in body swabs. In the remaining three farms (VIII, X and XII), the algae were identified in one (X; vagina and rectum), two (VIII; mouth and rectum) or three (XII; mouth, nose and vagina) control cows. The algae were recovered from 32.4% (12/37) of samples from different types of cow barn and equipment surfaces, 26.1% (6/23) of feed samples, 23.2% (13/56) of samples from watering troughs, 18.6% (8/43) of mud and soil samples and 7.5% (3/40) of manure samples (Table S3).

### *Prototheca* spp. identification

Upon molecular typing, all (166) *Prototheca* isolates recovered in this study were identified as *P. zopfii*, either genotype 1 or 2 and *P. blaschkeae* (Table S3). All but one (64/65; 98.5%) milk‐derived isolates belonged to *P. zopfii* gen. 2, and one isolate was *P. blaschkeae*. Of the *Prototheca* isolates found in body samples and stool, 32 (32/59; 54.2%) were *P. zopfii* gen. 2, 21 (35.6%) – *P. zopfii* gen. 1, and six (10.2%) – *P. blaschkeae*. *Prototheca zopfii* gen. 2 was more common than the two other species, in oral, vaginal and faecal samples, yet being slightly outnumbered by *P. zopfii* gen. 1 in nasal and rectal swabs. In 3 (27.3%) out of 11 CM/SCM cows with *Prototheca* algae found in their body swabs, the species/genotypes were the same as in milk samples. In four cows with *P. zopfii* gen. 2‐containing milk, *P. zopfii* gen. 1 was detected in rectal swabs (in one – also in vagina). In two cows with *P. zopfii* gen. 2 in their milk, *P. blaschkeae* was observed either in rectal or vaginal sample. One cow with mastitis due to *P. zopfii* gen. 2 and another one due to *P. blaschkeae* had *P. zopfii* gen. 1 and *P. zopfii* gen. 2 in their mouths, respectively.

The distribution of *Prototheca* spp. among environmental samples was similar to that observed for animal sites (Fig. 1B). The majority of environmental isolates were *P. zopfii* gen. 2 (20/42; 47.6%), followed by *P. zopfii* gen. 1 (14/42; 33.3%) and *P. blaschkeae* (8/42; 19.1%).

## Discussion

Mastitis is the most prevalent and economically burdensome health issue on dairy farms across Poland. On average, every second cow from a Polish farm develops mastitis at least once during the lactation period (Krukowski *et al*., 2009). Several locally based studies have reported consistent findings on the frequencies of major mastitis pathogens, showing, upon publication chronology, a steady decrease in the prevalence of *S. aureus* and *S. agalactiae* in parallel with an increase in the proportion of environmental pathogens such as streptococci, CNS, yeast‐like fungi and algae of the *Prototheca* genus (Krukowski *et al*., 2009; Wawron *et al*., 2010; Bochniarz *et al*., 2013). In Poland, bovine mastitis due to *Prototheca* algae was described for the first time in the early 2000s (Malinowski *et al*., 2002). However, no nationwide survey has ever been conducted, and the data currently available are limited to single herds or groups of herds within a single region. The present study is the first to attempt a comprehensive, large‐scale investigation into the epidemiology of *Prototheca* mastitis in dairy cattle in Poland, at a cross‐country level.

The mean within‐herd prevalence of protothecal mastitis was 10.2%, while the overall prevalence of the disease from this study was 8.3%. Both these values fall in the middle of those reported for dairy herds outside Poland, ranging from 5% up to 16.8% (Costa *et al*., 1996; Buzzini *et al*., 2004; Bueno *et al*., 2006; Shahid *et al*., 2016) Interestingly, the prevalence of *Prototheca* mastitis from this study was much higher than in most of the previously published reports from Poland, with the proportion of cows excreting *Prototheca* algae in their milk being usually below 0.5% (Krukowski, 2006; Krukowski *et al*., 2009). Only two recent studies have reported *Prototheca* incidences of 4.6% and 12.6% (Wawron *et al*., 2013; Jagielski *et al*., 2018). These differences can be explained by different geographical coverages of individual studies (three of four provinces with the highest prevalences of *Prototheca* mastitis had never been surveyed before) or sampling season (early spring and late summer are the periods of an increased abundance of the algae in the environment).

All but one *Prototheca* mastitis cases from this study were caused by *P. zopfii* gen. 2, which, congruently with previous studies (Gao *et al*., 2012; Shahid *et al*., 2016), confirms its role as a major aetiological agent. Whereas cases of *P. blaschkeae* infections, both clinical and subclinical, occur sporadically (Aouay *et al*., 2008; Marques *et al*., 2008; Jagielski *et al*., 2011), *P. zopfii* gen. 1 is considered non‐pathogenic and not involved in the aetiology of bovine mastitis. Yet, finding of 13 (30.9%) milk samples positive for *P. zopfii* gen. 1 in a study by Bozzo *et al*. (Bozzo *et al*., 2014) may not convincingly be explained only as the effect of environmental contamination. The pathogenic potential of *P. zopfii* gen. 1 cannot be excluded since this genotype produced subclinical infection of bovine mammary gland, upon experimental infection (Ito *et al*., 2011). As for the *Prototheca* in other than milk samples, the algae occurred most abundantly in faecal samples, which together with rectal swabs accounted for more than a half (59.3%) of all *Prototheca* isolates from cows (except those from milk). Most of the studies exploring the epidemiology of bovine *Prototheca* mastitis have shown faeces as an important reservoir of the algae (Ricchi *et al*., 2010; Shahid *et al*., 2016; Jagielski *et al*., 2018). Early works by Pore *et al*., which involved experimental feeding of laboratory animals with *Prototheca* spp., suggested that the algae transiently colonize the gastro‐intestinal tract of animals (Pore *et al*., 1983; Pore and Shahan, 1988). Finding, in this study, *Prototheca* spp. in other than faecal samples (i.e. nose and vagina) may indicate more extensive colonization, albeit contamination with faecal waste cannot be excluded.

Bovine mammary infections induced by *Prototheca* spp. are typically sporadic, with a subclinical course, which often progresses into a chronic condition, and a marked and prolonged elevation of SCC in milk. In this study, single cases of *Prototheca* mastitis occurred in four herds, whereas another four herds yielded multiple cases of the disease, with up to 12 animals affected. Multicase (≥ 2 case) outbreaks of bovine mastitis due to *Prototheca* spp. have been well described in the literature, with up to 34 cows per herd involved (Wawron *et al*., 2013). Close to 75% of the *Prototheca* mastitis cases found in this study were subclinical with a high SCC (mean SCC, 4.9 × 10^6^ cells ml^−1^). Significantly higher SCCs were observed for quarter milk of cows with CM (mean SCC, 9 × 10^6^ cells ml^−1^). These results are in line with earlier studies which documented protothecal infections as exclusively or predominantly subclinical (Shahid *et al*., 2016; Jagielski *et al*., 2018) with persistently high (> 10^9^ cells ml^−1^) SSCs (Jagielski *et al*., 2011, 2018; Wawron *et al*., 2013). However, several studies have described outbreaks of *Prototheca* mastitis with clinical manifestations only or in a vast excess over subclinical forms (Gao *et al*., 2012; Ricchi *et al*., 2013). Cases of *Prototheca* sp. infections with low SCC scores (< 2 × 10^5^ cells ml^−1^), although rare, have also been reported (Bueno *et al*., 2006; Pieper *et al*., 2012), urging for sampling also low SCC cows, preferably at individual‐quarter level, to avoid the dilution effect of uninfected quarters in a composite sample.

Among the environmental sources, the most *Prototheca*‐abundant was bedding, followed by barn walls, feed and drinking water. At all dairy farms, the proportion of *Prototheca*‐positive samples in these four categories ranged from 23.2% (water) to 35.3% (bedding), and the total isolation rate of *Prototheca* spp. from environmental sites was 21.1%. These results are similar to those from other studies, where the overall environmental prevalence of the algae ranged from 8.9% to 47% (Anderson and Walker, 1988; Osumi *et al*., 2008; Jagielski *et al*., 2018). *Prototheca* spp. could not be found in the cattle environment on three farms with ongoing mastitis, an observation reported also by other authors (Lagneau, 1996; Bueno *et al*., 2006; Ricchi *et al*., 2010). Conversely, five dairy farms, where no infected cows were identified, yielded *Prototheca*‐positive environmental samples. This is again what was observed in the past (Anderson and Walker, 1988; Jagielski *et al*., 2018). Whereas finding of *Prototheca* spp. in the dairy environment does not necessarily imply a mastitis problem within a herd, their absence in the surroundings of cows with protothecal infections can be attributed to insufficient sampling or long‐time persistence, with no shedding at the time of sampling.

It is noticeable that at the whole study level, no significant correlation (ρ < 0.4; *P *>* *0.3) between the numbers of *Prototheca* mastitis cases or bovine‐derived *Prototheca* strains and strains of environmental origin was observed. However, when farms without any *Prototheca* isolates from either animal or environmental sources were excluded, it was found that a higher isolation of *Prototheca* spp. from the environment translated into a higher isolation of the algae from milk and other animal sources (ρ > 0.8; *P *<* *0.03). This was however not observed if only strains of *P. zopfii* gen. 2 were analysed (ρ > 0.6; *P *>* *0.1).

Environmental samples, when viewed in terms of species affiliation, were most commonly contaminated with *P. zopfii* gen. 2 (47.6%), followed by *P. zopfii* gen. 1 (33.3%) and *P. blaschkeae* (19.1%). According to a very few studies which differentiated environmental *Prototheca* isolates, a clear predominance of either *P. zopfii* gen. 1 (73.4% or 90.6% of the isolates speciated; Osumi *et al*., 2008; Shahid *et al*., 2016) or *P. zopfii* gen. 2 (66.7% or 84.9%; Ricchi *et al*., 2010; Jagielski *et al*., 2018) was observed. Captivatingly, no other than the three *Prototheca* species (genotypes) could be isolated in this and other similar studies, indicating their predilection for different ecological niches.

Several case studies have repeatedly indicated that both farm management deficiencies, such as poor hygiene of housing, feeding and milking of cattle, and environmental conditions, including wetness, muddiness and large deposits of organic matter, are predisposing factors for the development and within‐herd dissemination of the disease (Janosi *et al*., 2001a,2001b; Bueno *et al*., 2006). However, in the only large‐scale study, which assessed farm‐level risk factors for *Prototheca* mastitis, no specific farm characteristics, farm management or failures in milking hygiene activities could be associated with an increased risk of the disease (Pieper *et al*., 2012). In this study, only size of farm and crew were correlated with the incidence of *Prototheca* mastitis (*P *<* *0.01; Cramer's *V* > 0.3); the larger was the farm acreage, a herd size or a number of employees, the higher was the incidence of mastitis.

Collectively, *Prototheca* mastitis appears as an emerging disease with still ill‐defined infection cycle. A high density of the algae in the herd environment may promote their dissemination and infectivity. Whereas episodic occurrences of *Prototheca* mastitis against an algae‐free environment may reflect long‐term, persistent or quasilatent infections, outbreak occurrences, under similar environmental conditions, may suggest a contagious mode of transmission. To resolve a question whether *Prototheca* spp. are more environmental or contagious mastitis pathogens or combine both these characteristics, further large‐scale investigations are required.

In this context, the authors of this study strongly emphasize the need for the development of an international network for the surveillance and control of *Prototheca* and other mastitis‐associated infections.

## Experimental procedures

### Study design and sample size

A cross‐sectional study design was applied with 16 dairy farms (I‐XVI), from each of the 16 administrative provinces of Poland (one farm per province), housing as many herds, varying in size from 19 to 584 cows, with a total of 2829 animals. The selection of the herds for the study was as follows. Up to five randomly selected dairy herds from each province were contacted and asked to participate in the survey, and the first that agreed, were included in the study. The farms (I–XVI) were inspected and sampled during a 3‐year period (i.e. between August 2015 and July 2018; Fig. S1). All farms had reported mastitis events over at least 2 months prior to the survey, according to the local veterinary records.

For estimating the apparent prevalence, the minimum sample size for the study was calculated using EpiTools epidemiological calculator (Sergeant, 2018), following the method described by Thrusfield (2005). The expected cow‐level prevalence was set at 4.6%, as this was the prevalence of *Prototheca* mastitis in a preliminary study carried out in Lublin province, Poland (Jagielski *et al*., 2018). Accordingly, the minimum overall sample size was computed to be 68. At the herd level, the minimum sample size calculated using EpiTools ranged from 15 to 61 (mean, 41.7 ± 15.7). As a rule, in herds up to 30 cows all animals were sampled. Whereas, 30, randomly selected, cows were sampled in herds whose size exceeded that number. This rule was a compromise between the values calculated by EpiTools and the numbers agreed by the farm owners. Any exceptions to this rule were due to the owners’ requests. Overall, a total of 400 lactating cows (range 14–30; mean, 25 ± 5.4 animals per herd) were included in the study.

A 25‐point questionnaire was developed and tested to collect basic information on the dairy farms and herds, in a standardized form (Table S1).

### Sample collection and clinical definitions

Udder quarter milk samples (QMS) were collected from lactating cows unless being treated or during their pre‐ and post‐partum periods. Clinical examination of the animals and milk quality assessment were performed, as described elsewhere (Jagielski *et al*., 2018). CMT was performed on foremilk QMS using the Mastirapid^®^ kit (Vetoquinol Biowet, Gorzów Wielkopolski, Poland), as per the manufacturer's instructions. The CMT results were scored as negative (0), trace (T), 1 + (=weak positive), 2 + (=distinctive positive) and 3 + (=strong positive; Quinn *et al*., 2002). CMT‐positive (score of T or above) quarter milk was sampled for culture. CMT‐positive QMS was also tested for somatic cell count (SCC) with an automated cell counter (Fossomatic 5000; FOSS Analytical, Hillerød, Denmark) and as described previously [32]. A threshold SCC of 2 × 10^5^ cells ml^−1^ was used for identification of mastitis (Jagielski *et al*., 2018). Clinical mastitis (CM) was defined as the presence of apparent changes in milk (mild), signs of inflammation in the udder (moderate), and/or generalized clinical symptoms (severe). Subclinical mastitis (SCM) was assessed based on the CMT and SCC scores (Amer *et al*., 2018).

Mastitis was microbiologically confirmed if at least one QMS was positive for microbial growth. If two or more QMS from the same cow yielded different pathogens, a multiple infection was considered. If more than one pathogen grew from a single QMS, a mixed aetiology was considered, with both organisms equally causative (Jagielski *et al*., 2018). Clinically healthy cows, negative upon CMT and culture were referred to as controls. From each dairy herd, two (herds II, IV), three (III, V, VI, VIII‐X, XII‐XV), four (VII), five (herds XI and XVI) or six (herd I) control animals were sampled for quarter milk, whole blood, faeces, and oral, nasal, rectal and vaginal swabs.

Cows whose milk yielded *Prototheca* spp. in culture were revisited and sampled for the same specimens as the control animals. Milk samples were collected following aseptic procedures, as described by the National Mastitis Council (2017). All other than milk samples, including environmental samples, were collected into sterile vials or containers. The samples were transported, under refrigeration (4°C), to the laboratory for microbiological evaluation. Blood samples were additionally subjected to standard haematological analysis.

The environmental sources sampled included drinking water from watering troughs (i), mud or soil, (ii) feed (feeders’ surfaces, forage, straw, hay), (iii) manure (manure corridors, collective manure chambers), (iv) bedding and (v) building and equipment surfaces (walls and floors of barns and feeding corridors, milking pipelines and teat cup liners) (vi).

Collection of other than milk samples, and environmental samples was sometimes impossible owing to the animal's behaviour, weather conditions or lack of permission of the farm owner.

### Primary isolation and culture

Samples from cows were inoculated on five types of culture media, namely Columbia Agar with 5% sheep blood (Oxoid, Thermo Fischer Scientific, Waltham, USA), McConkey agar (Sigma‐Aldrich, Saint Louis, USA), Sabouraud dextrose agar (SDA, Difco Laboratories, Detroit, USA), Edwards's LAB‐AGAR (BIOCORP, Warsaw, Poland) and *Prototheca* Isolation Medium (PIM; Pore, 1973). Cultures were incubated aerobically at 37°C for up to 96 h. Environmental samples were cultured in PIM only. Liquid and semisolid specimens (aliquots of 0.1 ml) were spread on the medium surface either directly, after dilution (ratio 1:1, w/v) in sterile water or after pre‐incubation in liquid PIM. Solid specimens were ground in a sterile porcelain mortar with sterile water and then plated as above. Swabs collected from animal body sites or farm facilities and equipment were streaked on agar plates. To enhance the detection and recovery of algae, and to prevent them from being overgrown by contaminating bacterial and/or fungal flora, the body site and environmental samples were enriched with liquid PIM (*ca*. 3–5 ml) and precultured for 48 h at 37°C, prior to plating.

### Species identification

A milk sample was considered culture‐positive if three or more bacterial and/or fungal colonies were detected, except for *Staphylococcus aureus* and *Streptococcus agalactiae* for which only a single colony was required. Contamination was suspected if a single sample grew, on the same culture plate, colonies of three or more different microorganisms (no such cases occurred in our study; Malinowski and Kłossowska, 2002). Macro‐ and micromorphology, Gram staining, haemolytic activity, catalase, oxidase and indol production, mannitol fermentation, esculin hydrolysis, slide and tube coagulase testing, and the Christie–Atkins–Munch–Petersen (CAMP) reaction were used to identify bacterial species. Presumptive yeast colonies were subcultured on SDA and confirmed upon microscopic examination.

Colonies suspected to be *Prototheca* spp. were subcultured on SDA and subjected to initial species identification. This included evaluation of colony and cellular morphology, and carbohydrate assimilation profiling, with the API 20C AUX system (Biomerieux^®^, Marcy‐l'Étoile, France). Phenotype‐based identification was confirmed by molecular methods, performed on total genomic DNA whose extraction procedure was detailed elsewhere (Jagielski *et al*., 2017). Species‐ (genotype‐)level identification was carried out using genotype‐specific PCR assays, as described by Roesler *et al*. (2006) and with a PCR restriction‐enzyme analysis (PCR‐REA) assay of partial *cytB* gene, as proposed by Jagielski *et al*. (2018).

All *Prototheca* strains, under appropriate species (genotype) assignations were preserved in Viabank™ vials (Medical Wire & Equipment, Corsham, UK) and stored at −70°C. The strains are part of the collection of the Department of Applied Microbiology, Faculty of Biology, University of Warsaw, and are available upon request.

### Statistical analysis

Two‐tailed Mann–Whitney *U*‐test for independent means was used to evaluate differences between SCCs and blood cell counts of mastitis and healthy cows. The associations between the health (case vs. control) or clinical status (CM vs. SCM) of the *Prototheca* mastitis cows and dairy farm characteristics were assessed using the chi‐square test with the Yate's correction, when necessary, or Fisher's exact test if the cell expected counts were < 5. The Cramer's *V* coefficient was used to determine the strength of any significant association. Correlations between the numbers (percentages) of *Prototheca* mastitis cases or bovine‐derived *Prototheca* strains and the number of strains recovered from the environment were evaluated using the Spearman rank correlation test. Statistical significance was set at *P *<* *0.05. All statistical analyses were performed using the ibm spss statistics (ver. 23) software package (IBM, Armonk, USA).

## Conflict of interest

None declared.

## Supporting information


**Fig. S1**. Prevalence of *Prototheca* spp. in dairy cows and their environment assessed on 16 (I‐XVI) dairy farms in Poland.

**Fig. S2**. A flowchart depicting the sampling strategy and results of sample culturing. Numbers in square brackets represent individual cows from which the samples were collected. *Within this number are seven cows with mixed protothecal‐bacterial (1) or yeast‐bacterial (6) mastitis infection.

**Table S1.** Farm management characteristics.
**Table S2.** Milk somatic cell counts and blood cell counts in cows with clinical and subclinical *Prototheca* mastitis and in control cows.
**Table S3.** Species‐ and genotype‐level identification of *Prototheca* isolates from this study.
