# Supplementary figures and images for: Prevalence of Prototheca spp. on dairy farms in Poland – a cross‐country study
Source: Microb Biotechnol. 2019 Mar 19;12(3):556–66. doi: 10.1111/1751-7915.13394 (PMC6465227; doi:10.1111/1751-7915.13394)

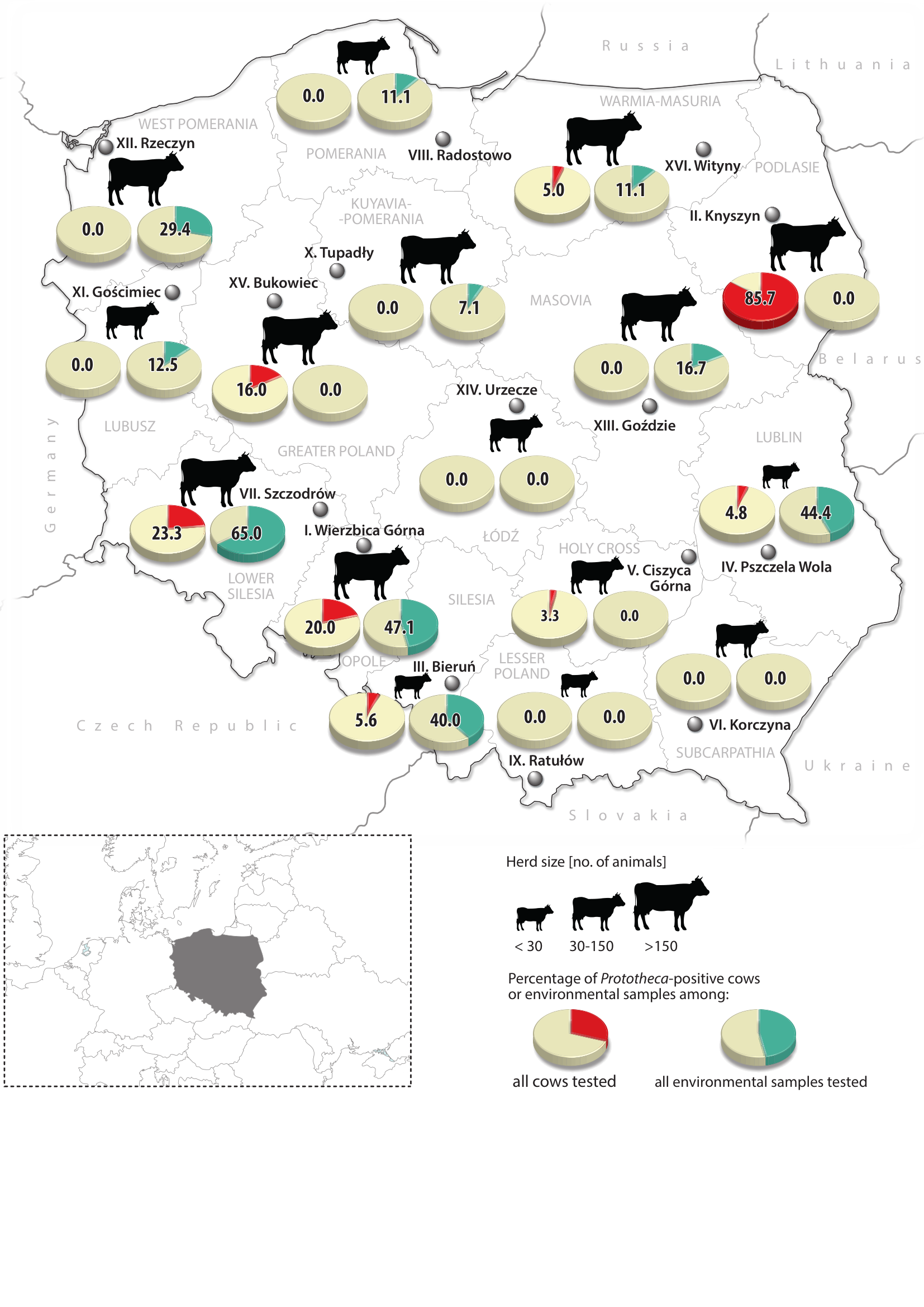

Supplement: Supplementary file 1 — Fig. S1. Prevalence of Prototheca spp. in dairy cows and their environment assessed on 16 (I‐XVI) dairy farms in Poland. [file MBT2-12-556-s001.tiff]

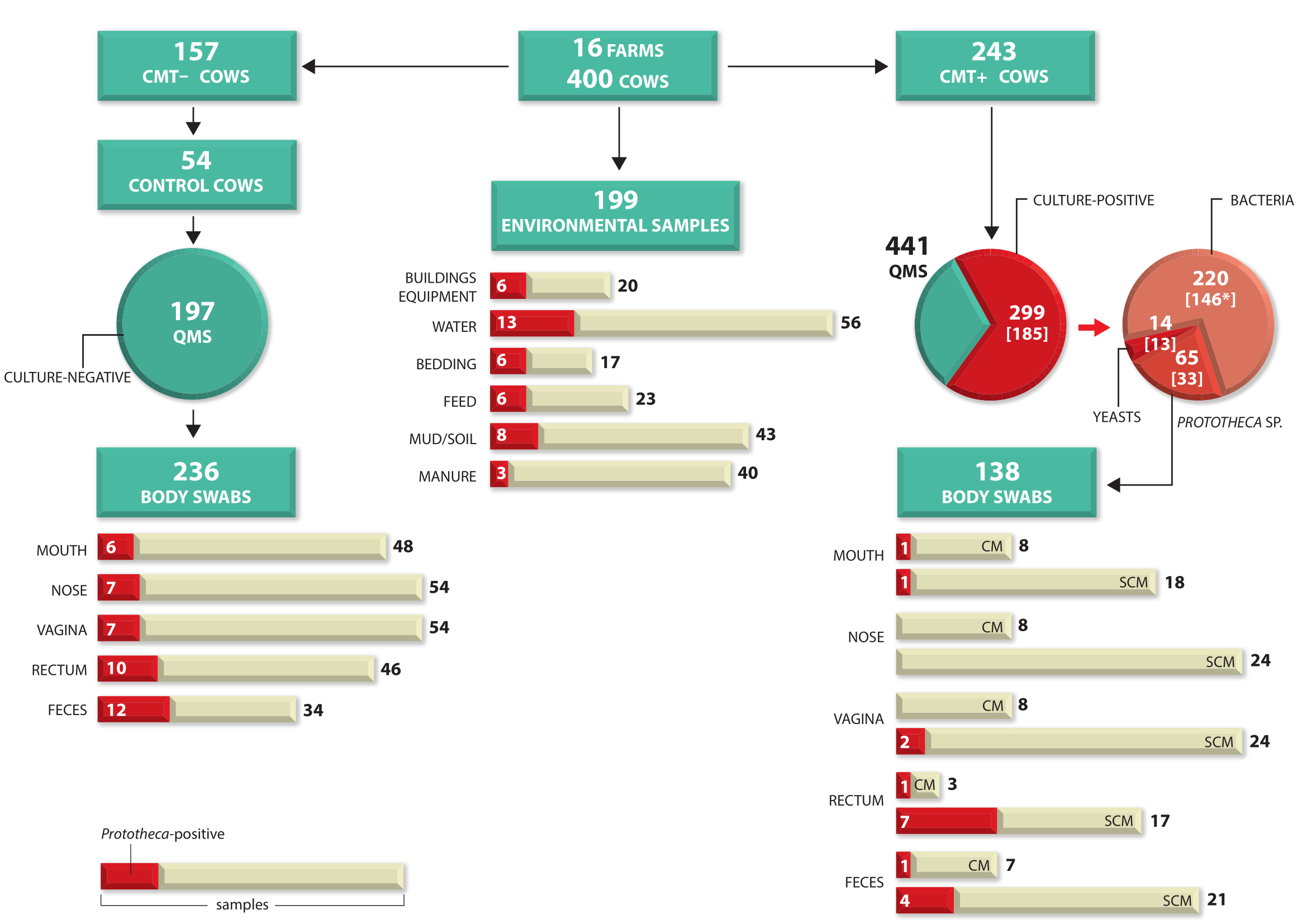

Supplement: Supplementary file 2 — Fig. S2. A flowchart depicting the sampling strategy and results of sample culturing. Numbers in square brackets represent individual cows from which the samples were collected. *Within this number are seven cows with mixed protothecal‐bacterial (1) or yeast‐bacterial (6) mastitis infection. [file MBT2-12-556-s002.tiff]
