# Supplementary material for: Prevalence of Prototheca spp. on dairy farms in Poland – a cross‐country study
Source: Microb Biotechnol. 2019 Mar 19;12(3):556–66. doi: 10.1111/1751-7915.13394 (PMC6465227; doi:10.1111/1751-7915.13394)
Supplement: Supplementary file 3 — Table S1. Farm management characteristics. Table S2. Milk somatic cell counts and blood cell counts in cows with clinical and subclinical Prototheca mastitis and in control cows. Table S3. Species‐ and genotype‐level identification of Prototheca isolates from this study. [file MBT2-12-556-s003.docx]

**Supplementary Table 1.** Farm management characteristics.

|  | Herd | | | | | | | | | | | | | | | |
| --- | --- | --- | --- | --- | --- | --- | --- | --- | --- | --- | --- | --- | --- | --- | --- | --- |
|  | I | II | III | IV | V | VI | VII | VIII | IX | X | XI | XII | XIII | XIV | XV | XVI |
| Herd size | 295 | 260 | 24 | 22 | 147 | 60 | 192 | 95 | 19 | 192 | 42 | 584 | 350 | 80 | 200 | 267 |
| Sampled animals^1^ | 6+6 (0/6) | 2+12 (5/7) | 3+1 (0/1) | 2+1 (1/0) | 3+1 (0/1) | 3+0 (0/0) | 4+7 (1/6) | 3+0 (0/0) | 3+0 (0/0) | 3+0 (0/0) | 5+0 (0/0) | 3+0 (0/0) | 3+0 (0/0) | 3+0 (0/0) | 3+4 (1/3) | 5+1 (0/1) |
| Cow breed^2^ | HF,MM,SM | HF,RW | HF | HF | HF | SM | HF | HF | RW | HF | HF,MO,SM | HF | HF | HF | MO | HF |
| Milk yield (L per year) | 2400000 | 2500000 | 168000 | 138000 | 1260000 | 420000 | 1400000 | 820000 | 86000 | 1880000 | 189000 | 7000000 | 3780000 | 840000 | 1440000 | 2240000 |
| Farm area^3^ | 590 | 585 | 40 | 115 | 260 | 120 | 400 | 270 | 10 | 425 | 120 | 1500 | 900 | 50 | 220 | 700 |
| Employees | 35 | 23 | 2 | 2 | 4 | 4 | 10 | 11 | 2 | 18 | 4 | 25 | 35 | 4 | 10 | 20 |
| Herd turnover | closed | open | closed | open | closed | open | closed | closed | closed | closed | open | closed | closed | closed | closed | closed |
| Slaughter^4^ (%) | 36 | 27 | 30 | 20 | 25 | 20 | 28 | 25 | 15 | 33 | 30 | 31 | 30 | 30 | 25 | 25 |
| Feeding^5^ | PMR | TMR | traditional | traditional | TMR | traditional | TMR | TMR | traditional | TMR | TMR | TMR | TMR | TMR | TMR | TMR |
| Housing | free stall | free stall | tie stall | tie stall | free stall | free stall | tie stall | tie stall | tie stall | free stall | tie stall | free stall | free stall | free stall | free stall | tie stall |
| Bedding (yes/no) | yes | yes | yes | yes | yes | yes | yes | yes | yes | yes | yes | yes | yes | yes | yes | yes |
| Bedding material | straw | sand | straw | straw | straw | straw | straw | straw | straw | straw | straw | straw | straw | straw | straw | straw |
| Body weight control | no | yes | no | yes | no | yes | no | no | no | no | yes | yes | yes | yes | no | yes |
| Veterinary examinations | yes | yes | yes | yes | yes | yes | yes | yes | yes | yes | yes | yes | yes | yes | yes | yes |
| Quarantine | no | yes | no | yes | no | no | no | no | no | no | no | no | yes | no | yes | no |
| Isolation | no | yes | yes | yes | yes | yes | yes | yes | yes | yes | no | yes | yes | yes | yes | no |
| Water quality control | yes | yes | yes | yes | yes | yes | yes | yes | yes | yes | yes | yes | yes | yes | yes | yes |
| Temperature control | no | yes | yes | yes | no | yes | no | yes | no | no | yes | no | yes | yes | yes | no |
| Humidity control | yes | no | yes | yes | no | no | no | yes | no | no | no | no | no | yes | no | no |
| Source of drinking water | municipal | municipal | municipal | municipal | municipal | municipal | municipal | municipal | municipal | municipal | municipal | municipal | municipal | municipal | municipal | municipal |
| Access to pasture or yard | yes | yes | yes | no | yes | yes | yes | yes | yes | yes | yes | yes | yes | yes | yes | no |
| Access to pond / river | no | no | no | no | no | no | no | no | no | no | no | no | no | no | no | no |
| Premilking teat disinfection^6^ | WDD | WDD | none | none | WDD | WDD | WDD | WDD | none | WDD | WDD | WDD | WDD | WDD | WDD | WDD |
| Postmilking teat disinfection | teat dip | teat dip | teat dip | none | teat dip | teat dip | teat dip | none | none | teat dip | teat dip | teat dip | teat dip | teat dip | teat dip | teat dip |
| Wearing of gloves | yes | yes | yes | yes | yes | yes | yes | yes | yes | yes | yes | yes | yes | yes | yes | yes |
| Wearing of coat | yes | yes | yes | yes | yes | yes | yes | yes | yes | yes | yes | yes | yes | yes | yes | yes |

^1^ Number of all animals subjected to milk and body-site culturing given in a format a+b, where a is a number of control cows and b is a number of mastitis cows; number of CM/SCM (clinical/subclinical) mastitis cases among mastitis cows were given in brackets (CM/SCM).

^2^ Holstein-Friesian (HF), Montbéliarde (MO), Polish Red (PR), Simental (SM), Polish Red-White (RW) cattle.

^3^ ha – hectares.

^4^ Percentage of animals segregated removed from herd for health or production reasons (average rate per year).

^5^ traditional – manual feeding, TMR – total mixed ration, PMR – partial mixed ration.

^6^ WDD – washing, drying, disinfection.

**Supplementary Table 2.** Milk somatic cell counts and blood cell counts in cows with clinical and subclinical *Prototheca* mastitis and in control cows.

|  | CM^3^ |  | SCM^4^ |  | Control |  |
| --- | --- | --- | --- | --- | --- | --- |
| milk SCC^1^, average ( x 10^6^/mL) | 9.1 ± 2.1 | *n* = 20 QMS^5^ | 4.9 ± 2.8 | *n* = 29 QMS | 0.2 ± 0.2 | *n* = 52 QMS |
| blood WBC^2^ ( x 10^9^/L)* | 9.4 ± 2.6 |  | 7.6 ± 1.6 |  | 7.6 ± 1.4 |  |
| lymphocytes ( x 10^9^/L)* | 3.2 ± 0.8 |  | 3.2 ± 1.0 |  | 4.3 ± 2.5 |  |
| neutrophils ( x 10^9^/L)* | 3.9 ± 1.3 |  | 3 ± 1.0 |  | 5.2 ± 4.0 |  |
| eosinophils ( x 10^9^/L)* | 1.9 ± 1.0 |  | 1 ± 0.6 |  | 1.3 ± 1.1 |  |
| cows, *n* (%)^6^ | 8 (2.1) |  | 25 (6.5) |  | 54 (14.1) |  |
| herds, *n* (%) | 4 (25) |  | 8 (50) |  | 16 (100) |  |

^1^ SCC, somatic cell count.

^2^ WBC, white blood cells.

^3^ CM, clinical mastitis.

^4^ SCM, subclinical mastitis.

^5^ QMS, quarter milk samples.

^6^ Percentage of cows (%) of different groups were given in brackets.

*, average counts for all animals from each group.

**Supplementary Table 3.** Species- and genotype-level identification of *Prototheca* isolates from this study.

|  | Total^*^ | Proto+^4^ | | *P. zopfii* gen. 1 | | *P. zopfii* gen. 2 | | *P. blaschkeae* | |
| --- | --- | --- | --- | --- | --- | --- | --- | --- | --- |
|  |  | *n* | % | *n* | % | *n* | % | n | % |
| QMS^1^ | 638 | 65 | 10.2 | 0 | 0 | 64 | 98.5 | 1 | 1.5 |
| BS^2^ | 374 | 59 | 15.8 | 21 | 35.6 | 32 | 54.2 | 6 | 10.2 |
| CM/SCM^*^ | 138 | 17 | 12.3 | 8 | 47.1 | 7 | 41.2 | 2 | 11.8 |
| mouth | 26 | 2 (1/1) | 7.7 | 1 (1/0) | 50 | 1 (0/1) | 50 | 0 (0/0) | 0 |
| nose | 32 | 0 (0/0) | 0 | 0 (0/0) | 0 | 0 (0/0) | 0 | 0 (0/0) | 0 |
| vagina | 32 | 2 (0/2) | 6 | 1 (0/1) | 50 | 0 (0/0) | 0 | 1 (0/1) | 50 |
| rectum | 20 | 8 (1/7) | 40 | 4 (0/4) | 50 | 3 (1/2) | 37.5 | 1 (0/1) | 12.5 |
| feces | 28 | 5 (1/4) | 17.9 | 2 (0/2) | 40 | 3 (1/2) | 60 | 0 (0/0) | 0 |
| control | 236 | 42 | 17.8 | 13 | 31 | 25 | 59.5 | 4 | 9.5 |
| mouth | 48 | 6 | 8.3 | 0 | 0 | 5 | 83.3 | 1 | 16.7 |
| nose | 54 | 7 | 13 | 4 | 57.1 | 3 | 42.9 | 0 | 0 |
| vagina | 54 | 7 | 13 | 1 | 14.3 | 5 | 71.4 | 1 | 14.3 |
| rectum | 46 | 10 | 21.7 | 6 | 60 | 3 | 30 | 1 | 10 |
| feces | 34 | 12 | 35.3 | 2 | 16.7 | 9 | 75 | 1 | 8.3 |
| ES^3^ | 199 | 42 | 21.1 | 14 | 33.3 | 20 | 47.6 | 8 | 19.1 |
| building and equipment surfaces | 20 | 6 | 30 | 3 | 50 | 2 | 33.3 | 1 | 16.7 |
| water | 56 | 13 | 23.2 | 6 | 46.2 | 6 | 46.2 | 1 | 7.6 |
| bedding | 17 | 6 | 35.3 | 3 | 50 | 2 | 33.3 | 1 | 16.7 |
| feed | 23 | 6 | 26.1 | 0 | 0 | 3 | 50 | 3 | 50 |
| mud/soil | 43 | 8 | 18.6 | 2 | 25 | 5 | 62.5 | 1 | 12.5 |
| manure | 40 | 3 | 7.5 | 0 | 0 | 2 | 66.7 | 1 | 33.3 |
| Total | 1211 | 166 | 13.7 | 35 | 21.1 | 116 | 69.9 | 15 | 9 |

* Numbers of isolates from clinical and subclinical (CM/SCM) cows were given in brackets in an “(a/b)” format, with “a” and “b” referring to isolates from CM and SCM cows, respectively.

^1^ QMS, quarter milk samples, collected from CMT-positive cows only (including CM/SCM cows and no control cows).

^2^ BS, body swabs.

^3^ ES, environmental samples.

^4^ Proto+, overall number (*n*) and percentage (%) of *Prototheca* spp. containing sample.
